# Envy and Counterproductive Work Behavior: The Moderation Role of Leadership in Public and Private Organizations

**DOI:** 10.3390/ijerph15071455

**Published:** 2018-07-10

**Authors:** Pilar González-Navarro, Rosario Zurriaga-Llorens, Adekunle Tosin Olateju, Lucía I. Llinares-Insa

**Affiliations:** 1 Research Institute of Personnel Psychology, Organizational Development and Quality of Working Life (IDOCAL), University of Valencia, Valencia 46010, Spain; Rosario.Zurriaga@uv.es; 2 Department of Psychology, College of Science, University of Canterbury, Canterbury 8041, New Zealand; adekunle.olateju@pg.canterbury.ac.nz; 3 Department of Social Psychology, Faculty of Psychology, University of Valencia, Valencia 46010, Spain; lucia.llinares@uv.es

**Keywords:** envy, counterproductive work behavior, leader-member exchange, public/private organizations, healthy organizations

## Abstract

Envy is a frequent emotion in work contexts where there is strong competition for resources and the leader is the person who manages them. When employees feel envy, they are likely to use counterproductive work behaviors (CWB), but the use of these behaviors may differ depending on the organization’s ownership. The goal of this study is to develop and test a model for the moderating role of Leader Member Exchange (LMX) in the relationship between envy and CWB in public and private organizations. The study design was cross-sectional. Data were collected from 225 Spanish employees in public and private organizations and analyzed using Path Analysis techniques. Results showed that envy was positively related to CWB, and that LMX was a significant moderator in the relationship between envy and CWB in public organizations, but not in private ones. However, this relationship is positive with high LMX, but less than in subjects with low LMX. Findings provide empirical support for the hypothesized conceptual model. This study is one of the first to explore LMX as a moderator of the relationship between envy and CWB. Thus, this study adds value to previous social exchange studies on LMX by integrating emotion research into the context of an exchange-based relationship. Our findings lead to several practical implications for creating healthy organizations.

## 1. Introduction

In today’s organizations, excellence is achieved not only through financial success, but also through physically and psychologically healthy human resources [1]. Currently, changes in the context are an inherent part of organizational dynamics. In this scenario, greater demands are placed on workers to adapt to new situations, and there is an increase in uncertainty [2]. These adaptation efforts have adverse effects on workers’ health, attitudes, and quality of working life [3]. In this regard, workplace health promotion is the best strategy to manage threatening and accelerating changes in the job context. However, scarcity and competition for resources produce high levels of envy. Envy is a universal emotion that damages relationships because it can result in spiteful behavior [4].

In fact, envy affects human relationships, generating hostility, competition between colleagues, or aggressive behaviors (see [5]). Envy is defined by Vecchio [6] as a “pattern of thoughts, emotions, and behaviors that result from an employee’s loss of self-esteem in response to a referent other’s obtainment of outcomes that one strongly desires” (p. 162). The goal of the envious person is to lower his/her level of envy by reducing the gap between the envious person and the envied person [7]. This can be accomplished by either moving oneself up or pulling the other person down. In fact, previous research proposes envy as an antecedent of counterproductive work behavior (CWB) [5,7,8]. In the workplace, CWB are defined as actions directed towards other members of the organization with the objective of hurting them through threats, nasty comments, and ridicule, or by damaging their performance [9].

The conception of envy as a predictor of CWB agrees with organizational researchers (see [10]). Studying discrete emotions (as envy) may increase our understanding of the role of emotions in organizational behavior and workplace management. The current state of the research on envy is not informative enough to address the dilemma that most current managers/leaders face in managing subordinates’ emotions, a situation where the quality of the relationship between leaders and followers (Leader Member Exchange—LMX) is important. It is well known that leaders can both high- and low-quality relationships with employees. A high-quality relationship involves the exchange of both material (e.g., tools) and non-material (e.g., social support) resources, whereas a low-quality relationship exclusively involves the exchanges described in the job position. The important role of LMX in employees’ outcomes has been demonstrated in the organizational literature. Most studies have focused on the relationship between LMX and positive employee behaviors [11]. However, few studies have addressed negative employee behaviors [12], and little attention has been paid to the role of LMX in employees’ envy [13]. It is important to fill this gap in order to understand the dynamics underlying the relationship between envy and CWB in the work context.

Successful healthy organizations require managers who are flexible in responding to organizational practices that may differ depending on the organization’s ownership [14]. The characteristics of the labor market in Spain (country where this research is carried out) are different from those of other countries in the European Union [15]. On the one hand, due to the financial crisis, private organizations have to engage in continuous restructuring and reduce the number of workers in order to be competitive. Long-term employment stability is not guaranteed, jobs are precarious, work hours are long (many hours of work), higher professional skills are required, and employees have to show greater commitment and a higher level of performance, etc. [16,17]. This new reality in private companies produces insecurities in workers due to fear of being unemployed [18]. On the other hand, Spanish public organizations offer stable and secure jobs. The selection procedure for public employees is based on the principles of equal merit and ability through examination. Once hired, the job lasts throughout their working life. In addition, the public sector has a system of predictable improvements, acceptable work conditions (for example, in the number of working hours and holidays, allowing employees to combine their work and personal lives), and possibilities of promotion and protection based on seniority in the workplace [17]. These conditions make the entire population want to work for the Spanish State. In public organizations, the salary remuneration does not change, and the relationship between workers and management is based on trust, unlike in private organizations, where it is based on increased performance [17]. 

As we can see, organizational ownership has an impact on the organization’s structure, processes, leadership styles, and managerial behaviors [19]. Hence, the management of these different ownership contexts can affect the relationship between psychological variables (e.g., envy at work) and work and/or organizational behaviors (e.g., CWB). However, little is known about the way emotions and management affect the organizational outcomes in public and private organizations. Management practices that do not consider these differences in ownership may be ineffective.

Thus, the main purpose of this study is to develop and test a model for the moderating role of LMX in the relationship between envy and CWB at work. We evaluate this model in public and private organizations. Our study makes a number of contributions to the literature. First, we extend the understanding of envy at work by exploring contextual factors (Leader Member Exchange—LMX—and ownership) that can influence this emotion and its consequences. In addition, we expand previous LMX literature by examining the organization’s ownership. Second, this study can be useful for managers, providing insight into the way their different relationships with employees can buffer negative emotions that often lead to counterproductive behaviors. Third, the greatest contribution of this study is the analysis of the same model, i.e., the moderator role of LMX in the relationship between envy and CWB, in public and private organizations. In this introduction, we will justify and elaborate the study hypotheses.

### 1.1. Envy and Counterproductive Work Behavior

Envy at work is a negative emotion that has been found at all organizational levels and in most cultures [20,21]. This emotion usually emerges at work due to competition for scarce resources, lack of time, or promotions. All of these factors have consequences for interpersonal relationships, reducing friendship bonds and damaging the employees’ exchange of knowledge. In addition, envy has been linked to mental health (e.g., depression and stress), generating health care costs in organizations. In fact, research has shown that more than fifty percent of organizations’ total costs are associated with poor health [22]. As Maris, Saideabadi, and Niazazari [23] state, envy has increased due to the current competitive work environment.

Therefore, envy has been understood as a common experience for most people, regardless of the context; that is, it is a relatively stable dispositional tendency or trait [21,24]. However, Crusius, Lange, and Cologne [25] conceptualize envy as a social functional emotion. Envy can be adaptive because it helps the individual to be aware of personal limitations in his/her social-work status and take corrective actions. Therefore, envy can occur when there is a precipitating event (e.g., personal and contextual features) and the employee perceives that a coworker has what s/he wants in order to improve his/her social standing [5]. According to Equity theory [26], individuals expect that the amount invested and gained in a relationship should be proportional to what another person invests and gains [27]. This theory further states that when individuals perceive inequalities, they may respond by attempting to raise the level of rewards received [28]. They can attempt to resolve the disparity by restoring equity through the expression of negative emotions. Thus, envy may be one of the main causes of widely documented behaviors that seek to undermine the reputation and job performance of others in the workplace [29]. Case studies have highlighted that envy engenders hostile behaviors ranging from incivility to physical aggression, both within the workplace and in other settings. Indeed, most research has shown that behavioral reactions to envy involve harming the other person [7]. A person who experiences envy not only focuses on wanting what the other person has, but also feels ill will toward him/her. The envious person might even consider removing or destroying the object of envy by using counterproductive work behaviors (CWB) [4].

CWB (e.g., sabotage, gossiping, and withholding vital information) are volitional behaviors intended to hurt or attempt to hurt organizations and/or organizational stakeholders, such as clients, co-workers, customers, and supervisors [30]. In order to understand why envy is a hostile emotion that often prompts CWB in the workplace, Hatampoor et al. [31] proposed that CWB arise to restore equity. In addition, Khan et al. [30] suggested that CWB are intended to eliminate or reduce the potential pain of comparison produced by envy. Hence, based on the aforementioned theoretical arguments and some empirical support, we hypothesize:

**Hypothesis 1** **(H1).** Envy will be positively related to counterproductive work behaviors.

### 1.2. Leader-Member Exchange in the Relationship between Envy and Counterproductive Work Behaviors

The relationship between supervisor and subordinate is an important determinant of partnership processes [25] and a healthy workplace [32]. Leader behavior can buffer the effect of envy on CWB [13]. Contemporary leadership proposals, such as transformational, servant, or authentic leadership theories, focus on the effects of leader behaviors on employee attitudes, motivation, and team outcomes, but none of them directly address the relationship between leaders and followers [33,34]. Nevertheless, according to the Leader Member Exchange (LMX) theory, the quality of the relationship between leaders and members is the key to understanding leaders’ effects on members, teams, and organizations. 

The LMX approach proposes that leaders are closer, friendlier, more inclusive, and more communicative with some members than with others. In other words, leaders have high quality, trusting, affective, and respect-based relationships with a subset of their team, whereas they tend to have a lower-quality exchange with other members [33]. 

The importance of this theory is that the leader’s ability to interact successfully with subordinates is crucial in maintaining effective organizations [35,36]. In fact, leaders act as gatekeepers of resources to support their employees [37]. However, because they have limited time and resources, leaders do not behave consistently toward all their subordinates [35,38,39]. This differential supervisory treatment leads to increased perceptions of inequalities and CWB [38,40,41]. Moreover, leaders treat subordinates differently depending on whether the subordinate is considered part of the “in-group” (high-quality relationship) or the “out-group” (low-quality relationship) [40]. 

Moreover, in the organizational context, leaders are decision makers, and some of their decisions might be perceived as unfair. When employees perceive that they are being treated worse than others by their supervisor, this differential supervisory treatment can lead to increased perceptions of inequalities and CWB [40]. Previous research has reported a negative relationship between LMX and deviant behaviors (see [42]). This relationship has been explained by Bolino and Turnley [43] using relative deprivation theory. As these authors state, employees with low-quality LMX may exhibit deviant behaviors as a reaction to relative deprivation (i.e., when a discrepancy occurs between the way things are and the way things should be, the person feels tension that can be described as relative deprivation).

The relationship between envy and LMX is complex. Organizational literature has shown that LMX can elicit envy in the workplace (e.g., [12,13]). In addition, some studies analyze how envy affects interpersonal processes such as LMX [1], whereas others analyze envy’s possible association with a behavioral response, and whether this relationship can be affected by the relationship with the supervisor [5]. In this research, we are interested in discovering to what extent having a high or low quality relationship with the leader modifies the relationship between envy and CWB. Although the relationship between envy and CWB is widely accepted in the literature, we propose that a high-quality relationship with the leader can lead to a reduction in counterproductive behavior, even if envy continues to exist. That is, when an employee feels envious because a fellow employee has something s/he wants, a relationship of trust and respect with the leader will make it easier for the envious employee to exhibit less spiteful behavior.

We consider two mechanisms to explain how LMX reinforces or decreases CWB performed by envious employees. First, high-quality LMX encourages more interactions with the supervisor, and this situation minimizes any negative behavior responses [44]. Second, high-quality LMX can change deviant behaviors because it reduces the relative disadvantage envious employees feel in their comparisons with envied coworkers [45]. In this regard, envy leads to more hostile behavior when supervisors create a competitive relationship between coworkers [46]. Thus, we are interested in the way LMX influences the relationship between envy and counterproductive work behavior. We are not interested in dispositional antecedents of envy, but rather its relationship with leadership and its effects on CWB. We seek to capture the role of LMX and its influence on the relationship between envy and counterproductive work behavior in a sample of Spanish workers. From a practical perspective, understanding the mechanisms connecting these two variables can be useful for designing strategies to manage envy at work [12]. Based on the aforementioned arguments, we hypothesize the following:

**Hypothesis 2** **(H2).** Leader-member exchange will moderate the relationship between envy and CWB.

### 1.3. Leader-Member Exchange, Envy, Counterproductive Work Behavior, and Public and Private Organizations

Managers operate under different and very specific constraints in private and public organizations [14]. Therefore, we think it is important to study the moderating role of LMX in the relationship between envy and CWB in different organizational ownership contexts. 

We explore the relationships among envy, LMX, and CWB in the public and private sectors, a topic that has not been previously explored, to the best of our knowledge. The literature finds that the leader’s style is related to ownership. Therefore, the relationship model proposed in this study probably functions differently depending on the type of ownership of the organization. In order to understand how this relationship occurs, there is a need for studies that test the same relationship in public and private organizations.

The characteristics of public and private organizations are related to sources of funding/capital and management-control mechanisms that distinguish the management practices of leaders in each type of organization [19]. In fact, both public and private organizations intend to achieve quality products or services and generate benefits [43]. Nevertheless, public and private organizations show differences in their missions, values, strategic objectives, and the implementation of high-performance work practices, such as recruitment and selection, teamwork, rewards and recognition, and performance management [47]. Likewise, organizational politics are present in both types of organizations. Previous research [48] has reported significant differences in perceptions of organizational politics of public sector and private sector employees. Public sector employees consider their environment to be more political [49]. Additionally, in the public sector, there is more job security and, consequently, public sector employees want to keep their jobs. Although public employees are aware of the political climate, they “become part of the political climate and merge in it” ([50] p. 127). Vigoda-Gadot and Beeri [50] state that LMX is more important in a highly political organizational culture (i.e., public sector) because high-quality LMX can help employees to overcome difficulties in interpersonal relationships. In public organizations, exchange relationships between leader and follower are not limited to material transactions; they may also include social exchanges of psychological benefits (i.e., trust, respect), whereas there is less need for LMX when the environment is less political (i.e., private sector). In these contexts, relationships between managers and employees are less relevant because private organizations are less focused on values and achieving consensus among opposing groups, and more focused on economic benefits. Along these lines, Hassan and Hatmaker [51] point out that, despite the limitations and restrictions in the public context, LMX is a key factor influencing employees’ behaviors. Likewise, Audenaert et al. [52] emphasize that employees who perceive high-quality LMX feel safe enough to discuss problems in public organizations. 

Surprisingly, few studies have examined the roles of LMX and organizational ownership (e.g., [48,53,54]), or they have focused mainly on private management and less on public management [25]. Moreover, we did not find any studies that analyzed envy or CWB in two sectors, even though organizational ownership can be a factor that influences the relationship between envious employees and their envied co-workers [45].

As Crusius et al. [25] state, from a social-functional approach to envy, the assessment of the social nature of envy has been underestimated, and little is known about its relationships with other social variables (e.g., LMX and ownership). Leader behavior can be used to maintain and establish hierarchical structures and the organizational culture, and understanding how leader behavior works in different sectors (public-private) is a crucial question. Moreover, the literature has traditionally focused on dispositional envy, and other social variables (e.g., LMX) have been analyzed as antecedents; however, there are no studies about differences between public and private organizations as contextual elements that could affect this relationship. This social perspective of envy allows us to highlight the moderating role of LMX in the increase or decrease in CWB by envious employees in different contexts. It is necessary to fill this gap. Based on the aforementioned arguments, and given that management in public organizations is highly politicized [55], we hypothesize:

**Hypothesis 3** **(H3).** Leader-member exchange will moderate the relationship between envy and CWB in public organizations. In private organizations, this relationship will be attenuated or nonsignificant. In any case, the occurrence of CWB will be lower when envious employees report higher levels of LMX.

**Figure 1:**
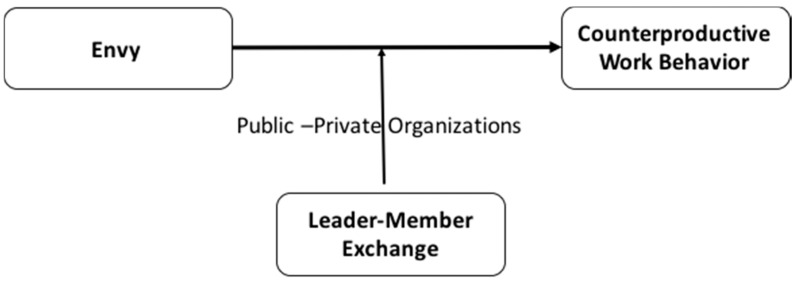
Hypothesized conceptual model.

Therefore, the goal of this study is to develop and test a model of the moderating role of LMX [6] in the relationship between envy and CWB at work. We evaluate this model in public and private organizations (see Figure 1). We use LMX theory and measurement because LMX captures the quality of the relationships between leaders and subordinates, and research has revealed its usefulness in contexts of change such as the current one [56,57]. Although the relationship between envy and CWB has been studied (e.g., [7]), knowledge about the moderating role of the quality of the relationship with the leader in this relationship is scarce. Specifically, we argue that an envious employee can perform fewer CWB if he/she has a high-quality relationship with the leader because the leader is responsible for distributing the resources. However, when the envious worker has a low-quality relationship with the leader, he/she is more likely to feel incapable of improving the situation and try to reduce the distance from the envied party through counterproductive behaviors.

## 2. Materials and Methods

### 2.1. Participants and Procedure

The study design was cross-sectional. The sampling procedure used was incidental purposive sampling [57]. The purposive sampling technique involves deliberately choosing a participant due to the qualities s/he possesses. We used this sampling technique because in this research it was necessary to select an equivalent number of employers working in public and private organizations. The conditions for participating in the study were to be currently working and not hold a manager position. Data were collected from 2015 to 2016 through self-report questionnaires completed voluntarily by the participants at the workplace in the presence of the researcher, after they had provided their informed consent. Participants received instructions and information about the procedure for filling out the questionnaire, and the researcher helped to resolve and explain any doubts that arose. The researchers stressed that anonymity and confidentiality were guaranteed, that there were no right or wrong answers, and that participants should answer the questions as honestly as possible [58].

A total of 225 Spanish workers (48% men and 52% women) in different organizations were surveyed. The mean age of respondents was 37.87 years (SD = 10.9). These workers came from the public (*N* = 104) and private (*N* = 121) sectors. None of the participants had a managerial role. Table 1 presents the description of both samples.

### 2.2. Measures

The following questionnaires were used.

#### 2.2.1. Envy

First, envy was measured using the workplace envy scale developed by Vecchio [59]. This scale consists of five items rated on a five-point Likert-type scale (1: strongly disagree to 5: strongly agree). High scores indicate higher levels of envy, which means that the participant feels that his/her efforts are not valued, that someone else has better job assignments, and that s/he is the underdog at work. A sample of an item is: “It is somewhat annoying to see that others have all the luck in getting the best assignments”. The Cronbach α coefficient for this scale is 0.80 in this sample. 

#### 2.2.2. Interpersonal Counterproductive Work Behavior

Second, participants rated the extent to which they engaged in interpersonal counterproductive work behaviors on a 12-item scale [7]. High scores indicated that they performed actions to obstruct or interfere with the work of others, harm the reputation of others, withhold information about work, or speak badly about others in order to hurt them, etc. The items included behaviors such as “interfere with X’s performance”, “try to sabotage X’s reputation”, “withhold work-related information from X”, or “create coalitions against X”. Participants were asked to rate each item on how accurately it represented actions they engaged in toward others, using a rating scale ranging from 1 (strongly disagree) to 5 (strongly agree). The Cronbach α coefficient for this scale is 0.89 in this sample. 

#### 2.2.3. Leader Member Exchange

Third, leader member exchange was measured using seven items with a four-point Likert-type scale (1: always/completely to 4: never/not at all) developed by [60]. High scores indicate high LMX quality, which means that the relationship is based on mutual trust, respect, and obligation between supervisors and employees. Examples of items are: “To what extent do you think your boss can understand your problems and needs?”; “How would you describe your relationship with your boss?” The scale shows high reliability, with a Cronbach α coefficient for this scale of 0.89 in this sample.

#### 2.2.4. Sociodemographic Variable

Fourth, three sociodemographic variables were measured: (a) age (we followed Martin’s typology [61] to establish three age groups −20–39, 40–49, over 50); (b) sex; and (c) educational level (compulsory education, post-16 education, and higher education). 

All the questionnaires were translated from English into Spanish by two bilingual translators. The two translations were then back-translated into English by another translator. The translators compared their results item by item to assess the equivalence of the Spanish translation. If there were discrepancies, the items were discussed and revised until reaching a consensus. Finally, the comprehensibility of the translated items was assessed by subject matter experts. Moreover, to mitigate common method variance (CMV), which had created false internal consistency in the answers on the questionnaires [58], we used a different scale format and anchor points [12].

### 2.3. Data Analysis

Initial descriptive statistics, bivariate correlations between all variables, *t*-tests, and ANOVA were examined. To test the hypotheses, path analysis was then conducted using the maximum likelihood (ML) estimation method. Given the small sample size (*N* = 225; Sample 1 *N* = 104; Sample 2 *N* = 121) and the number of parameters to be estimated, we modeled relationships among observed (not latent) variables. We tested a moderated model that included all the study hypotheses. This model proposed the moderating effect of leader-member exchange in the relationship between envy and CWB. The path analysis approach allows the hypothesized relationships to be examined. This analytical technique facilitates the investigation of direct and indirect effects between variables [62]. The indirect effects involved in the model were tested using the bias corrected (BC) bootstrap confidence interval method [63].

A variable was created with envy and LMX together as a moderating variable [64]. First, we tested the hypothesized relationship using path analysis in the entire sample. Second, multi-group comparison was employed to test the model for this study in public and private organizations separately. Third, path analysis was carried out in public organizations separately. ML parameter estimates were calculated. Model fit was assessed with a combination of fit indices [65]. The goodness of-fit indices for the model were evaluated using absolute [66] and relative fit indices [67]. Specifically, four fit indices were employed: (a) the χ^2^ statistic with Satorra–Bentler correction and χ^2^/df < 2 [68], a nonsignificant chi square indicates good model fit; however, chi square is sensitive to sample size [66]; (b) the Comparative Fit Index (CFI) and the Normed Fit Index (NFI) [69]; and (c) the Root Mean Square Error of Approximation (RMSEA) [70]. The CFI compares the sample covariance matrix with a null model that assumes that all the latent variables are uncorrelated (null/independence model). CFI values above 0.90 indicate a good fit to the data and adequate model fit. The RMSEA is a measure of the average size of the fitted residuals per degree of freedom. Values of about 0.05 or less indicate a close model fit, values of about 0.08 or less indicate a fair model fit, and values <0.1 indicate poor fit. NFI assesses the model by comparing the χ^2^ value of the model to the χ^2^ of the null model. Bentler and Bonnet [71] recommend values above 0.90 to indicate a good fit. This fit indicates that the hypothesized relations in the model are plausible. If the initial model offers a poor fit to the data, the second step is to modify the model.

Combinations of descriptive and inferential statistics were calculated with the Statistical Package for the Social Sciences (SPSS), version 22, and Analysis of Moment Structures (AMOS), version 22. Sample size is adequate, correlations between variables are not high, 0.85 [66], and the sample data follow a standard normal distribution [72].

## 3. Results

### 3.1. Desciptive and Preliminary Analysis

Means and standard deviations for the studied variables were calculated (Table 2), as well as Pearson bivariate correlations. There were moderate positive correlations between LMX and CWB and envy. Higher levels of envy were also found to be concurrently associated with more CWB. In the two samples in this research, there were no statistically significant differences in the study variables based on sex, age, and educational level. Therefore, these control variables were not included in the model. Moreover, there were significant differences in envy between the public and private sector samples (M_Public_ = 2.17 vs. M_Private_ = 2.45, t_(5,223)_ = 2.33, *p* = 0.02).

### 3.2. Test of Hypothesized Model

Figure 1 shows the tested model, and Table 3 presents the goodness of fit indices and chi-square difference tests. The model presents an acceptable fit to the data, and the correlations are significant in the total sample. These results provide support for Hypothesis 1: Envy predicts CWB. The hypothesized moderating role of LMX in the relationship between envy and CWB (H2) was tested, and the results support it.

**Figure 2:**
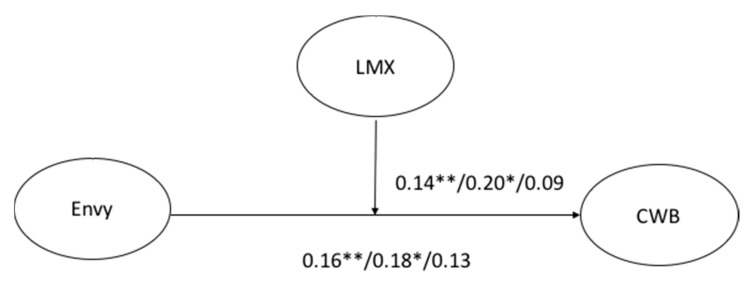
Results of path analysis for the model in the total sample, public sector, and private sector. Note: LMX = Leader Member Exchange; CWB = Counterproductive Work Behavior.

Later, we examined whether there were differences between the public and private sectors. The multi-group analysis showed adequate levels of model fit (Table 3). However, the results revealed that the relationship between envy and CWB was not moderated by LMX in the private sector. In the public sector, the moderated results were significant, indicating that LMX moderates the relationship between envy and CWB. Table 4 shows the standardized coefficients for the impact of the model on CWB. This result supports hypothesis 3: LMX moderates the relationship between envy and CWB differently in public and private organizations. Thus, because the model was not significant for the private sector, we did not estimate whether the model was identical or equivalent to the constrained model across groups [69]. Figure 2 shows the results of the path analysis.

**Figure 3:**
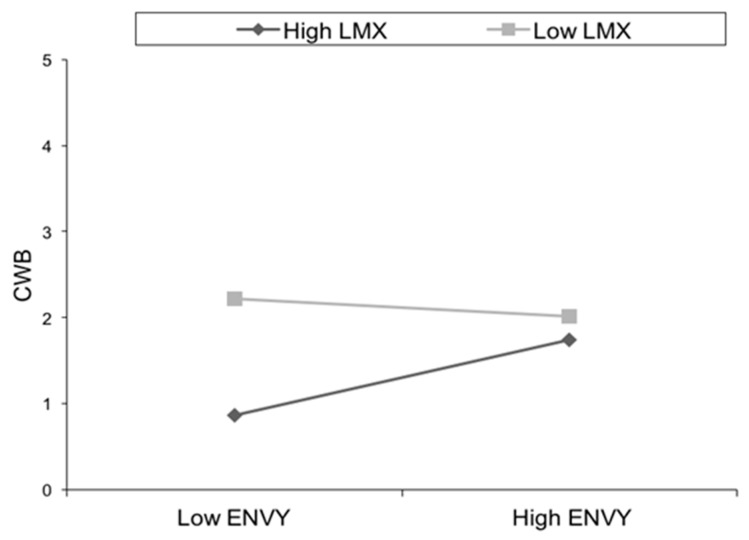
Interaction effects of LMX and envy on CWB. Note: LMX = Leader Member Exchange; CWB = Counterproductive Work Behavior.

Finally, the model was estimated in the public organization sample. Table 3 shows the goodness of fit indices, which indicate that LMX moderates the relationship between envy and CWB in the public sector (b = 0.20, *p* < 0.05). The estimators (Table 4) indicate that all the estimated variables correlate adequately as well. To further interpret the interaction effect proposed in Hypothesis 3, and following Aiken and West [73] and Dawson [74] we computed simple slopes for high and low values of the moderator. Figure 3 shows that LMX moderates the relationship between envy and CWB because this relationship is different in the presence of high (+1SD) and low (−1SD) LMX. The results show that the slope for high LMX is significant (Effect = 0.31; SE = 0.10; t = 2.96; *p* < 0.01), but it is not significant with low LMX (Effect = 0.001; SE = 0.91; t = −0.002; NS). There is a positive direction of the relationship between envy and CWB with high LMX, contrary to our predictions. However, as Figure 3 reveals, CWB is always lower with high LMX than with low LMX. Then, we used *t*-tests to analyze whether employees with high envy have significant differences in CWB depending on LMX. The results show that there are significant differences (t = 6.42; ρ = 0.001). If LMX and envy are high, CWB increases, but there is significantly less CWB than in subjects with low LMX. In the same direction, if LMX is high and envy is low, *t*-tests show less CWB than in employees with low LMX (t = 3.24; ρ = 0.01). Thus, LMX is important because envy leads to less CWB when LMX is high. In sum, our results point to the moderator role of LMX, although in our study LMX does not buffer the effects of envy in the expected way. Thus, hypothesis 3 is only partially confirmed.

## 4. Discussion

Leader membership exchange is a key factor in healthy organizations [8]. Moreover, emotions are closely related to employees’ well-being. However, the role of the leader in the management of employees’ emotions and their possible consequences is an underexplored issue [6]. This study investigated the moderating role of LMX in the relationship between envy and counterproductive work behaviors in organizations. In addition, the present study considers the role of the ownership of the organization (public versus private) in this relationship because it is an essential political factor that has an impact on management efficiency.

The results reveal that envy predicts counterproductive work behavior. This result agrees with previous research [31,33,34] and, consistent with Equity Theory [26], increases the understanding of the relationship between envy and CWB. Thus, when an employee feels envy, s/he is motivated to engage in negative behavior. Research has shown that reducing employees’ CWB guarantees a healthy organization [4]. Therefore, it is important to minimize CWB at work.

Moreover, in this study, LMX moderated the relationship between envy and CWB. This result supports our second hypothesis and highlights the role of LMX in the relationship between envy and CWB. However, as mentioned above, the relationships among envy, LMX, and CWB are complex. Several studies have supported the relationship between LMX and envy at work [5,12,13,24]. Some research analyzes the relationship between supervision and CWB [42,43]. However, the moderator role of LMX in the relationship between envy and CWB has not received much attention in previous research. This result is important from a practical point of view. Leaders can contribute to managing the negative emotions of their employees by having a high quality relationship with them, thus reducing the negative consequences of these emotions.

Additionally, we found that LMX operates differently depending on the specific ownership of the organization. In this study, the moderator role of LMX in the relationship between envy and CWB occurs in public organizations, but not in private ones (Hypothesis 3). In public organizations, as the literature indicates [7,22,29], the relationship between envy and CWB is positive, but there is less CWB than when the quality of the relationship with the leader is worse. In this regard, a possible explanation is that LMX attenuates the positive association between envy and CWB. A high quality relationship with the leader implies having access to more and better information [75] (i.e., promotion opportunities). Therefore, when the perception of the quality of the relationship with the leader is higher, the more envious employee can perceive more support and access to resources, and so the association between envy and CWB can be positive, but attenuated. Another possible explanation for the role of LMX in the relationship between envy and CWB can be formulated based on the betrayal framework [76]. Following Elangovan and Shapiro [77], betrayal occurs when there is a violation of the norms and expectations of a relationship. In this context, when there is a high quality relationship with the leader, the breach of the envious employee’s expectations can be considered a betrayal, and as a result, more CWB may appear. Future studies would be needed to clarify the moderator role of LMX in the relationship between envy and CWB.

In the private sector, LMX is not a moderator of the relationship between envy and CWB. One possible explanation for this difference would be the Spanish financial crisis from 2008 to the present, which has particularly affected the private sector. Private organizations are controlled by market forces, and their main aim is to increase performance [17]. Job instability, unemployment, job mobility, ease of dismissal, and other conditions resulting from the crisis have led to changes in labor and social relationships at work. In this situation, managers in the private sector may focus more on solving everyday labor problems than on having high-quality relationships with employees. The public sector, however, has been affected less by the economic crisis because it is basically controlled by political authorities [11]. It offers stable and secure jobs, and so managers can focus not only on benefits, but also on human resources practices. 

Although some authors [19] suggest that the public sector would gain efficiency and improve performance by using the management techniques of the private sector (see e.g., Alfort & Greve [78]; Shi, Chon, Liu, & Ye [79]), this paper highlights that focusing on human resources, rather than exclusively on efficiency, is a useful management tool to maintain the quality of work life and improve well-being. For example, an important attribute that differentiates managers in public organizations from managers in private organizations is the decision-making process and the way conflicts are handled. In this regard, Schwenk [80] suggests that managers in the private sector see conflict as a negative sign because it indicates that some members of the organization do not believe that the results of the strategic action are positive. However, for managers in the public sector, the conflict present in a strategic decision has a positive component because it shows that different stakeholders participate in the process, ensuring that the final decision will represent, or at least consider, their interests.

Likewise, Nutt [81] shows that public managers value the use of advisory practices when making decisions, whereas private managers prefer the use of analytical practices. One explanation for this difference may lie in the social mission of managers in their organizations. The ultimate goal of public managers is to maximize collective value and relationships. By contrast, managers in private organizations seek to maximize the wishes of the organization’s shareholders and, therefore, prioritize economic considerations [48,82].

## 5. Limitations and Implications

This study has some potential limitations. First, self-report measures were used, which can cause common error bias [7,28,83], but this can be justified by the nature of the variables considered in this study. However, the use of one survey is considered a valid option when ‘both the predictor and criterion variables are capturing an individual’s perceptions, beliefs, judgments, or feelings’ [84]. Moreover, to mitigate common method variance (CMV), we used a different scale format and anchor points [12]. Second, it would be interesting to investigate high- and low-quality LMX relationships and their effects over time. Future longitudinal designs would make it possible to obtain information about the dynamics of this relationship. Third, the findings obtained have limited generalizability, due to the use of an incidental purposive sampling. Etikan et al. [57] state that this sampling method is useful for this type of study, but it is not truly representative of the population because of the subjective way of choosing the sample. However, the sample represents an important sector of workers from public and private organizations in Spain. Future studies should analyze these relationships in other countries and consider the quality of employment (i.e., type of contract).

This research contributes to the literature on leadership, envy, and counterproductive work behavior in the following ways. First, this study is consistent with previous research that proposes envy as a predictor of CWB [7,8,85]. In addition, it is one of the first studies to explore Leader-Member Exchange as a moderator of the relationship between envy and CWB in public and private contexts. Moreover, the findings support and expand the social exchange perspective of LMX, which is consistent with previous research [7,12,86]. Furthermore, our findings reveal the negative side of social exchanges between leader–follower dyads [87]. The social exchange perspective shares a similar central tenet of equity and LMX theory, emphasizing the reciprocal nature of workplace relationships. Therefore, when an envious employee perceives that his/her relationship of trust with the supervisor is breached, s/he might reciprocate in a manner that is counterproductive for the well-being of the organization and its members. Furthermore, when the work relationships between the envious person and the envied person involve the exchange of information, help, and coordination, the envious person might withhold these resources from the envied party, hampering his/her work performance. The exchange of incorrect information would slow down productivity and degenerate into interpersonal conflict, with some negative effects that would affect the organization as a whole [7]. In this regard, leadership training courses to develop high quality LMX could improve behaviors designed to reduce negative consequences of envy and create healthy organizations. In summary, this study adds value to previous social exchange studies on LMX by integrating emotion research in the context of an exchange-based relationship [88].

Our findings lead to several practical implications for public managers. These managers can create a healthy organizational climate and avoid situations that lead to envy in their daily interactions with employees. In this regard, managers should implement transparent and fair policies when assigning tasks and monitoring work processes [42]. Another strategy managers can use to decrease envy would be to establish clear performance objectives, which may also increase feelings of organizational fairness [88]. Managers also need to pay close attention to existing employees with low LMX relationships by showing frequent interest in them. To increase the quality of LMX relationships, managers should establish informal meetings or social activities with the workers. Managers can also reduce employees’ CWB in the organization through the creation of grievance handling and a settlement framework that can immediately resolve an employee’s complaint and negative feelings. In sum, public managers should engage in behaviors that foster high LMX as much as possible. 

## 6. Conclusions

In order to create healthy organizations, it is necessary to reduce the negative consequences of employee envy. The leader plays an important role in moderating the effect of envy on CWB at work. In our study, this moderation occurs in the public sector, but not in the private sector. In a financial crisis like the recent one, managers in the public sector can focus more on human resources practices than on benefits, whereas managers in the private sector have to focus more on profits.

## Figures and Tables

**Table 1:** Sample Characteristics.

	Public Sector (*N* = 104)			Private Sector (*N* = 121)		
	%	Mean	SD	%	Mean	SD
Men	41.30			53.70		
Women	58.70			46.30		
Age		41	9.96		35	10.88
*Highest Educational Level Attained*						
-Elementary School	5.60			8.30		
-Professional Training	16.50			23.10		
-Bachelor/Higher Education	77.90			68.60		
*Occupations*						
-Trade and marketing	6.70			25.60		
-Administration and Management	26.90			24		
-Cultural and Community Services	22.10			6.60		
-Health	13.50			5.80		
-Tourism				5.80		
-Computers and Communication	4.80			2.50		
-Mechanical Manufacturing				1.70		
-Other sectors	26			28		

**Table 2:** Descriptive Statistics and Bivariate Correlations.

Variable	Total Sample				Public Sector				Private Sector			
	M	SD	2	3	M	SD	2	3	M	SD	2	3
1. CWB	1.73	0.67			1.67	0.69			1.78	0.66		
2. Envy	2.32	0.89	0.23 **		2.17	0.90	0.26 **		2.45	0.86	0.19 *	
3. LMX	2.80	0.63	−0.35 **	−0.27 **	2.86	0.64	−0.38 **	−0.25 **	2.76	0.62	−0.31 **	−0.27 **

Note: *N* = 224. * *p* < 0.05; ** *p* < 0.01. LMX = Leader Member Exchange; CWB = Counterproductive Work Behavior. M = Mean; SD = Standard Deviation.

**Table 3:** Summary of Path Analysis.

Model	χ^2^	df	*p*	Δχ^2^	Δdf	Critical Value	NFI	GFI	CFI	RMSEA
Total sample (*n* = 225)	4.72	2	0.09				0.92	0.99	0.95	0.08
Multi-group Unconstrained	6.11	4	0.19				0.90	0.99	0.96	0.50
Multi-group Constrained	7.37	7	0.49	1.25	3	0.74	0.88	0.98	0.99	0.01
Public Sector (*n* = 104)	1.47	2	0.48				0.96	0.99	1	0.00

Note: χ^2^ = chi-square; df = degrees of freedom; *p* < 0.05; *p* < 0.01; Δχ^2^ = chi-square differences; Δdf = differences in degrees of freedom; NFI = Normed Fit Index; GFI = Goodness of Fit Index; CFI = Comparative Fit Index; RMSEA = Root Mean Square Error of Approximation. LMX = Leader Member Exchange; CWB = Counterproductive Work Behavior.

**Table 4:** Standardized Path analysis effects.

Variables	Total Sample		Multi-Group Analysis				Public Sector	
			Public Sector		Private Sector			
	Est.	*p*	Est.	*p*	Est.	*p*	Est.	*p*
Envy	0.16	0.001	0.18	0.04	0.13	0.15	0.18	0.04
LMX	−0.29	0.001	−0.32	0.001	−0.27	0.001	−0.31	0.001
LMX Moderator of envy	0.14	0.01	0.20	0.02	0.09	0.30	0.20	0.02

Note: Estimate = Beta value; *p* < 0.05; *p* < 0.01; LMX = Leader Member Exchange; CWB = Counterproductive Work Behavior.
